# Parinaud Syndrome in a Patient With Microangiopathic Lesions in the Bilateral Gangliocapsular Region and Left Thalamus

**DOI:** 10.7759/cureus.58120

**Published:** 2024-04-12

**Authors:** Renu Magdum, Priyanka S Aher

**Affiliations:** 1 Ophthalmology, Dr. D. Y. Patil Medical College, Hospital and Research Centre, Pune, IND

**Keywords:** parinaud syndrome, microangiopathies, convergence-retraction nystagmus, lid retraction, upgaze palsy

## Abstract

Parinaud syndrome, which most commonly involves the dorsal midbrain, has classical features of upward gaze paralysis, convergence-retraction nystagmus, and pupillary light near dissociation. A 62-year-old male presented to the Eye department with diminution of vision and symptoms of dry eye with associated difficulty in walking. Examination revealed nystagmus while performing convergence test. An MRI revealed lesions in the thalamic and gangliocapsular regions. Microangiopathies involving the thalamus and gangliocapsular region can lead to Parinaud syndrome. In our case, microangiopathies were most probably hypertensive.

## Introduction

Assessment of pupillary light reflex is an integral part of ocular examination. Knowledge of the pupillary pathway is essential for its localizing value. The afferent pupillary fibers start from the retinal photoreceptors and terminate in the pretectal nucleus of the midbrain. Further synapses with the Edinger Westphal nucleus of the third nerve nuclear complex and their further journey to the ciliary ganglion and finally the sphincter pupillae complete the reflex [1].

In the region of the dorsal midbrain, the pupillary fibers lie in proximity to the vertical gaze center. The midbrain center for the near reflex is probably situated more ventrally than the pretectal nucleus. Therefore, lesions involving the dorsal internuncial neurons involved in the light reflex spare the near reflex fibers until later [1]. Lesions such as infarcts, demyelination, tumors, or trauma-related lesions may give rise to a characteristic clinical picture that was first described by Henri Parinaud in 1800 and bears his name.

We report a case of Parinaud syndrome secondary to thalamic infarcts attributable to hypertensive and diabetic microangiopathy.

## Case presentation

A 62-year-old diabetic (type 2 diabetes mellitus) and hypertensive male presented to the Eye outpatient department with complaints of reduced vision for far and near since three months, which was insidious in onset and gradually progressive in nature. The patient also had dryness of the eyes since one month and had a history of dysarthria and imbalance while walking. There was no complaint of diplopia. He also had a history of spectacle use for far and near vision since 10 years.

**Figure 1:**
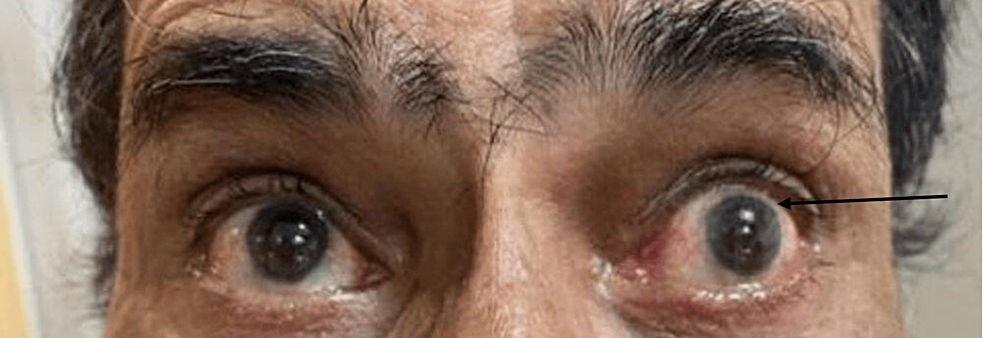
Image showing difficulty in conjugate upward gaze and retraction of left upper eyelid while looking upwards.

When the patient was trying to look up with both his eyes open, upward lid retraction and superior scleral show was seen in both eyes, with the left eye more than the right eye (Figure 1).

**Figure 2:**
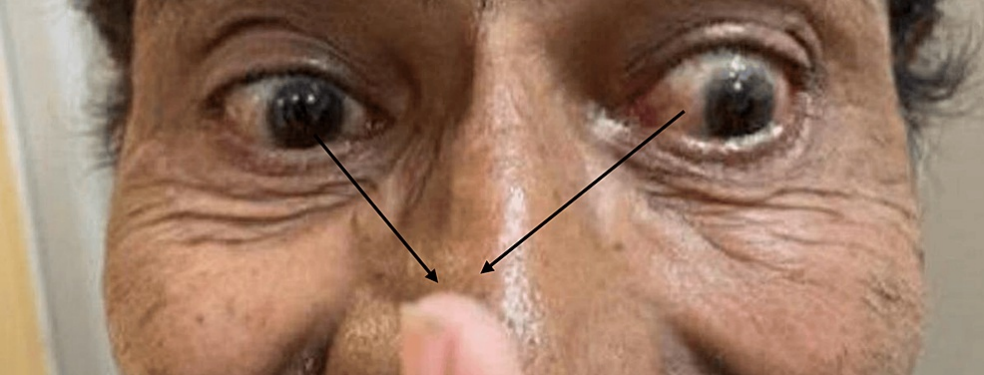
Image showing convergence weakness when the patient tries to fixate towards the fingertip (left eye shows more convergence weakness than the right eye).

No proptosis was present. On the convergence test, there was inability to converge. On trying to converge, patient showed nystagmus. Gaze-evoked nystagmoid movements were noted on the left side. Inability to converge is shown in Figure 2, where the patient is asked to fixate toward the fingertip.

Pupils were slightly dilated and showed impaired pupillary light reaction, while the accommodative pupillary response was intact (light near dissociation).

On the Hirschberg test, 15-degree exotropia was noted in the left eye, which was taking and maintaining fixation on cover test. On cover-uncover test, alternating exotropia in both eyes was noted.

Vision was corrected to 6/12 in the right eye and 6/36 in the left eye with refraction. His near vision improved to N10 with refraction in both eyes. He had immature senile cataract with grade 2 nuclear sclerosis in both eyes. Fundus showed no pathology.

On examination, corneal sensation was reduced in both eyes in all quadrants. There was poor Bell's phenomenon in both eyes and 0.5 mm lagophthalmos in the left eye. Dysdiadochokinesia was also present with tandem gait.

On general examination, the patient was conscious, cooperative, and well-oriented to time, place, and person. On examination, his pulse and blood pressure were noted to be within normal limits. Random blood sugar levels were within normal limits.

Thyroid function test was performed to rule out thyroid ophthalmoplegia, which came out to be within normal limits. The patient’s symptoms did not improve on ice pack test, which ruled out myasthenia gravis.

**Figure 3:**
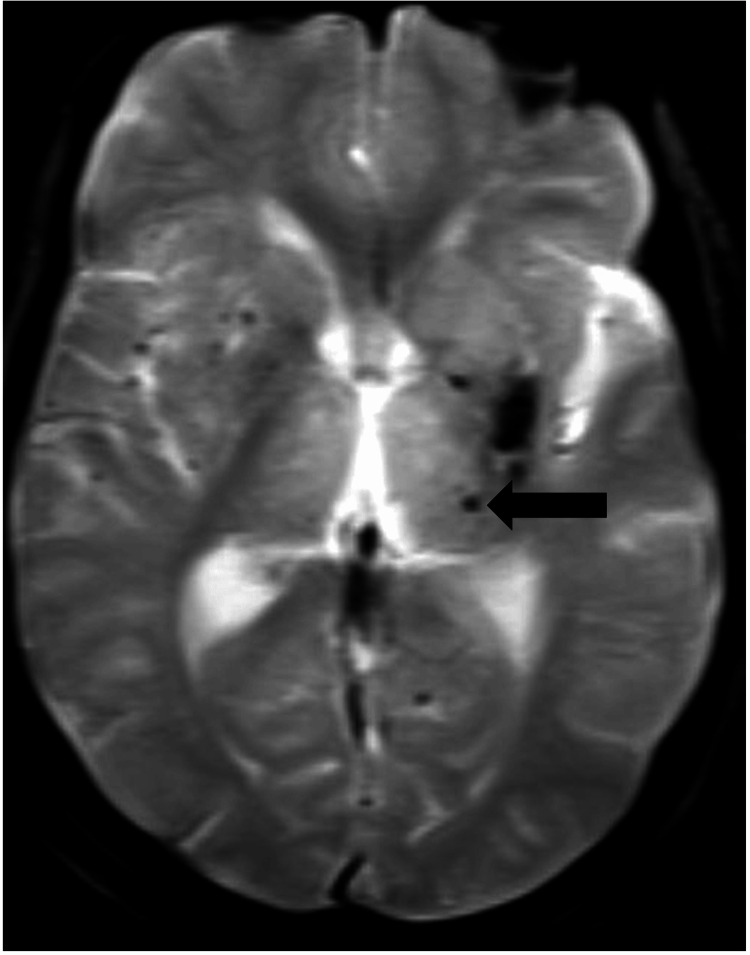
Blooming noted in MRI, suggestive of microhemorrhages in the thalamus (as shown by the black arrow).

MRI of the brain was performed, which revealed thalamic lesions in the form of microhemorrhages, most probably due to hypertensive microangiopathy (Figure 3).

## Discussion

Parinaud syndrome is also called Sylvian aqueduct syndrome, pretectal syndrome, dorsal midbrain syndrome, Koerber-Salus-Elschnig syndrome. The site of this syndrome lies in the dorsal midbrain at the level of the superior colliculus. It is classically described by the triad of upward gaze paralysis, convergence retraction nystagmus, and pseudo-Argyll Robertson pupil (also known as light-near dissociation). Patients may complain of difficulty in looking upwards, decreased near vision, double vision (in 65% cases), oscillopsia, and associated neurological symptoms [2]. Commonly reported symptoms are diplopia, blurred vision for near, and difficulty in looking up. Diplopia has been described as a presenting symptom, but our patient had no complaints of diplopia. The patient can present with ataxia as a symptom or the practitioner can notice it as a sign. The patient might describe it as not being able to co-ordinate in day-to-day activities.

In a report of 40 cases with Parinaud syndrome by Shields et al., 7.5% of them presented with ataxia [3]. In patients with Parinaud syndrome, there is also a presence of Collier's sign (eyelid retraction in primary gaze). Most patients have a limitation of conjugate upward gaze due to involvement of vertical gaze centers located at the posterior commissure. When the patient tries to converge, irregular jerky nystagmus is seen, and when the patient tries to look upwards, eyelid gets retracted. This is known as convergence retraction nystagmus. Pupillary involvement shows a light near dissociation with poor response to light but brisk response with convergence. The crossing fibers between the pretectal nucleus and Edinger Westphal nuclei of both sides are vulnerable to compression by mass lesions compared to near reflexes fibers, which are more ventral. Minor elements are a pseudo abducens palsy, convergence, and accommodative insufficiency.

In a similar case reported by Menon et al. in 2007, a 30-year-old man with thrombosis of the deep cerebral venous system had features of Parinaud syndrome along with bilateral papilledema [4]. In this case, multiple thalamic infarcts were noted due to microangiopathies, most commonly due to hypertensive and diabetic microangiopathy in this case.

Other cases reported in the literature are mostly of pineal gland tumors compressing the superior colliculi posteriorly accompanied by signs and symptoms of raised intracranial pressure.

A case of reverse Parinaud syndrome was reported by Menon et al. [5], where there was restricted downgaze instead of upgaze in a patient operated for a pineal gland tumor. Similar cases with traumatic and vascular pathologies in anterior mesencephalon have been reported in the literature by Cogan et al. and Jacobs et al. [6,7].

## Conclusions

This is a case report of a patient with microangiopathies involving the thalamus and the gangliocapsular region causing Parinaud syndrome. A patient presenting with upgaze paralysis, convergence weakness, and pseudo-Argyll Robertson pupil associated with abnormal gait should undergo investigations to rule out neurological causes. Even when fundus examination was normal, microhemorrhages were found in the thalamic region.
